# Identification of small molecule inhibitors targeting FGFR through molecular docking-based screening

**DOI:** 10.3389/fonc.2026.1733391

**Published:** 2026-01-29

**Authors:** Weibo Hou, Kun Liu, Ping Wang, Kefan Zheng, Qingran Kong, Xinyu Wang, Qi Zhao, Liang Cheng

**Affiliations:** 1 College of Bioinformatics Science and Technology, National Health Commission Key Laboratory of Molecular Probes and Targeted Diagnosis and Therapy, Harbin Medical University, Harbin, Heilongjiang, China; 2 Department of Oncology, Heilongjiang Academy of Traditional Chinese Medicine, Harbin, Heilongjiang, China; 3 Zhejiang Provincial Key Laboratory of Medical Genetics, Key Laboratory of Laboratory Medicine, Ministry of Education, School of Laboratory Medicine and Life Sciences, Wenzhou Medical University, Wenzhou, Zhejiang, China; 4 West China Second University Hospital, Sichuan University, Chengdu, Sichuan, China; 5 Institute for Regenerative Medicine, Shanghai East Hospital, Shanghai Key Laboratory of Signaling and Disease Research, School of Life Sciences and Technology, Tongji University, Shanghai, China; 6 Department of Cardiology, Ningbo Medical Center, The Affiliated LiHuiLi Hospital of Ningbo University, Ningbo, Zhejiang, China

**Keywords:** CRC, FGFRs, molecular docking, molecular dynamics simulation, small molecule inhibitors

## Abstract

**Background:**

FGFRs genetic alterations such as mutations, amplifications, and chromosomal translocations are prevalent in cancers, leading to the initiation and progression of tumors by enhancing FGFR signaling. The substantial problems arising from the lack of decisive clinical evidence have resulted in the cessation of some inhibitor applications, and identifying effective small molecule inhibitors that selectively target FGFRs can advance the therapy of cancers driven by FGFRs abnormalities.

**Methods:**

The three-dimensional structure of the FGFR1/2/3/4 protein and the amino acid positions within the tyrosine kinase domain were downloaded from the PDB database, and small molecule data were extracted from the ZINC15 database. Then, we used molecular docking and dynamics simulations to assess compounds interacting with FGFR proteins, and screening potential small molecules targeting FGFR. Finally, we evaluated its effects by two CRC cell line HCT116 and NCI-H716.

**Results:**

In the study, by docking with 2.8 million small molecules, we identified three promising FGFR small molecule inhibitors ranked in the top average absolute difference in free energy. By evaluating the binding stability of the docking pose of the three compounds, we found that ZINC000101867325 could form the stable binding interactions with FGFR1/2/3. And, ZINC000101867325 inhibited the activity of FGFR signaling, and resulted in cell apoptosis and decrease in cell proliferation and migration in colorectal cancer cell lines. In addition, ZINC000101867325 is also predicted to target FGFR2 mutations in colorectal cancer patients.

**Conclusion:**

We predicted three small molecules targeting FGFRs, and ZINC000101867325 shows superior chemical bond types and stability with FGFR1/2/3, and inhibits FGFR signaling in CRC cell lines. This study provides novel FGFRs inhibitors, which enrich treatment strategies for cancers.

## 1 Introduction

Fibroblast growth factor receptors (FGFRs) are a group of transmembrane receptors with tyrosine kinase activity that activate cellular signaling pathways through the binding of fibroblast growth factor (1). The activation plays a crucial role in the regulation of biological functions such as cell differentiation, growth, and proliferation (2). In humans, the FGFR family consists of four highly conserved receptors (FGFR1/2/3/4), with three extracellular immunoglobulin-like domains, a transmembrane domain, and two intracellular tyrosine kinase domains (3, 4). Genetic alterations such as mutations, amplifications, and chromosomal translocations are prevalent in various cancers (5–7). These alterations enhance the activity of FGFR signaling, which contributes to the initiation and progression of tumors.

The oncogenic potential of FGFR genetic alteration has driven the development of numerous small molecule inhibitors that target FGFRs (8). The small molecule inhibitors approved by the US Food and Drug Administration (FDA) for treating tumors harboring FGFR alterations include erdafitinib, pemigatinib, infigratinib, and futibatinib (8–12). Although research on small molecule drugs targeting FGFRs genetic alterations in various cancers is extensive, early clinical trials have revealed significant variability in the efficacy of anti-FGFR treatments among patients with different FGFRs abnormalities (8, 13). The significant challenges in short of definitive clinical trials lead to the discontinued applications of some inhibitors, such as infigratinib. Consequently, the identification of novel small molecule inhibitors is necessary for the therapy of cancers driven by distinct FGFRs genetic variations.

Using the high-performance computers, drug virtual screening has become efficient and validated, comparing to experimental high-throughput screening for hit identification and optimization (14–16). Computer-aided method is used to discover, develop, and analyze drugs and active molecules with similar biochemical properties (17–19). The elucidation of protein crystal structures provides a wealth of high-quality three-dimensional structural information, which is crucial for drug development. Concurrently, the AlphaFold2 model could accurately predict residue conformations, greatly improving the efficiency of drug research and development (20). In this study, we docked approximately 2.8 million small molecule drugs to the crystal structures of FGFR1-4, and predicted three small molecules targeting FGFRs. Among them, ZINC000101867325 shows superior chemical bond types and stability with FGFR1/2/3, and inhibits FGFR signaling in CRC cell lines. Homology modeling analysis also revealed that ZINC000101867325 is predicted to interact with mutated FGFR2. This study underscores the potential of identification of novel active chemotypes through structure-based screening.

## 2 Results

### 2.1 Screening of potential FGFR ligands

**Figure 1:**
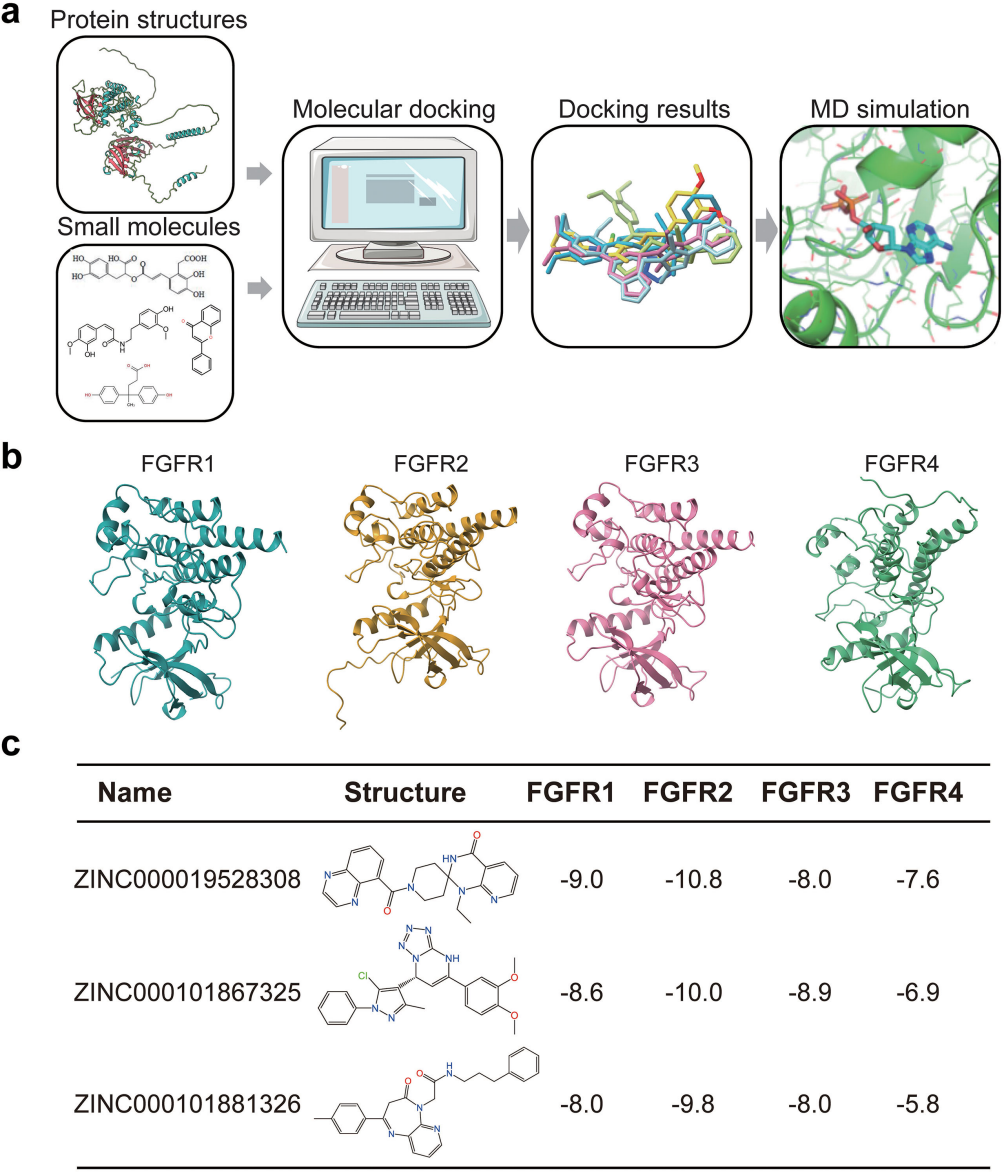
Screening of novel FGFR ligands. **(a)** Molecular docking for new small molecule ligands for FGFR proteins. **(b)** Intracellular tyrosine kinase domains of FGFR1/2/3/4. **(c)** Binding free energy of three potential and novel FGFR ligands.

To identify novel FGFR inhibitors, we conducted molecular docking and dynamics simulations to assess compounds interacting with FGFR proteins (**Figure 1a**). To comprehensive understand the FGFRs structures, we compared the AlphaFold2-predicted structures with the FGFRs crystal structures. While the FGFR crystal structures lack details on flexible residues, such as FGFR1 (**Supplementary Figure S1a**), the AlphaFold2-prediction provides the high-confidence three-dimensional structural information. Previous studies indicate that small molecule inhibitors targeting FGFR predominantly bind the intracellular tyrosine kinase domains (TK domains) (21, 22). Thus, we extracted the TK domains of FGFR1–4 from the PDB database with specific amino acid sequences (FGFR1: 464–765; FGFR2: 456–768; FGFR3: 454–767; FGFR4: 472–761), and by AlphaFold2, we predicted the TK domains of FGFR1-4 (**Figure 1b**). Utilizing AutoDock Vina for semi-flexible molecular docking, we analyzed optimal binding conformations of 2.8 million small molecules from the ZINC15 database with the TK domains, ranking them by the group mean absolute difference in binding free energy (23). For the molecular docking process, the TK domains of FGFRs were fixed as the receptor structure, allowing only the small molecules to adjust their conformations. We added polar hydrogens to the cleaned receptor, and set respective docking box centers for FGFR1-4. We prioritized potential FGFR-targeting molecules based on: i) Selecting the best of nine possible docking postures for each compound; ii) Ranking the average absolute differences in free energy; iii) Ordering the docking free energies from lowest to highest.

Ultimately, we identified three small molecule compounds as potential FGFRs inhibitors, ZINC000019528308, ZINC000101867325, and ZINC000101881326 (**Figure 1c**; **Supplementary Figure S1b**). Their docking free energies with the FGFRs were all below -5 kcal/mol, and ranked in the top three for average absolute difference in free energy. Notably, the interaction between these compounds and the FGFR2 TK domain showed the lowest binding free energies compared to FGFR1, FGFR3, and FGFR4, suggesting a potential specific inhibitory to FGFR2.

### 2.2 Molecular dynamics simulation of the potential FGFR ligands

To elucidate the binding stability of these three potential FGFR ligands, we conducted molecular dynamics simulation on the complexes formed by the docking of ZINC000019528308, ZINC000101867325, and ZINC000101881326. We performed the Root Mean Square Deviation (RMSD) trajectories and fluctuations for the small molecules and FGFRs TK domains over the full simulation period (**Supplementary Figures S2a, b**), and found that the RMSD for FGFR1 and the three molecules stabilized after 50 ns, maintaining around 0.3 nm until the simulation’s conclusion. The structures of FGFR2, FGFR3, and FGFR4, along with the small molecules, generally remained stable within a 0.2 nm fluctuation range for most of the simulation, suggesting overall structural stability for FGFR1–4 binding with the ligands. ZINC000101867325 and FGFR4 displayed four notable fluctuations: initially unstable, causing an increase in RMSD to 0.3 nm, followed by fluctuations stabilizing around 0.38 nm at about 70 ns, and a final increase in the last 5 ns. The overall fluctuation remained under 0.5 nm, indicating stability in the simulated molecular structures. The potential small molecule ligands targeting FGFRs reduced the radius of gyration, stabilizing between 1.95 and 2.15 nm with a fluctuation amplitude under 0.2 nm (**Supplementary Figure S2c**).

The solvent-accessible surface areas (SASA) of the complexes between the four small molecules and the FGFR protein fluctuated between 150 and 190 nm², stabilizing over time (**Supplementary Figure S2d**). The root mean square fluctuation (RMSF) was calculated to measure each atom’s deviation from its average position during the simulation, with higher values indicating more intense atomic movements. The small molecule ligands and the known FGFR inhibitor TAS-120 (24) both showed RMSF values for the largest amino acid residue binding to the FGFRs under 1.8 Å, suggesting minimal internal motion and relative stability of the complex structures (**Supplementary Figure S2e**). The three compounds exhibited characteristics similar to those of the known inhibitor TAS-120, highlighting their potential as drug compounds. Additionally, each TK domain of the four FGFRs contains only a single histidine residue, and this histidine does not participate in binding with these small molecules (**Supplementary Figure S3**), so we did not perform a protonation selection process.

**Figure 2:**
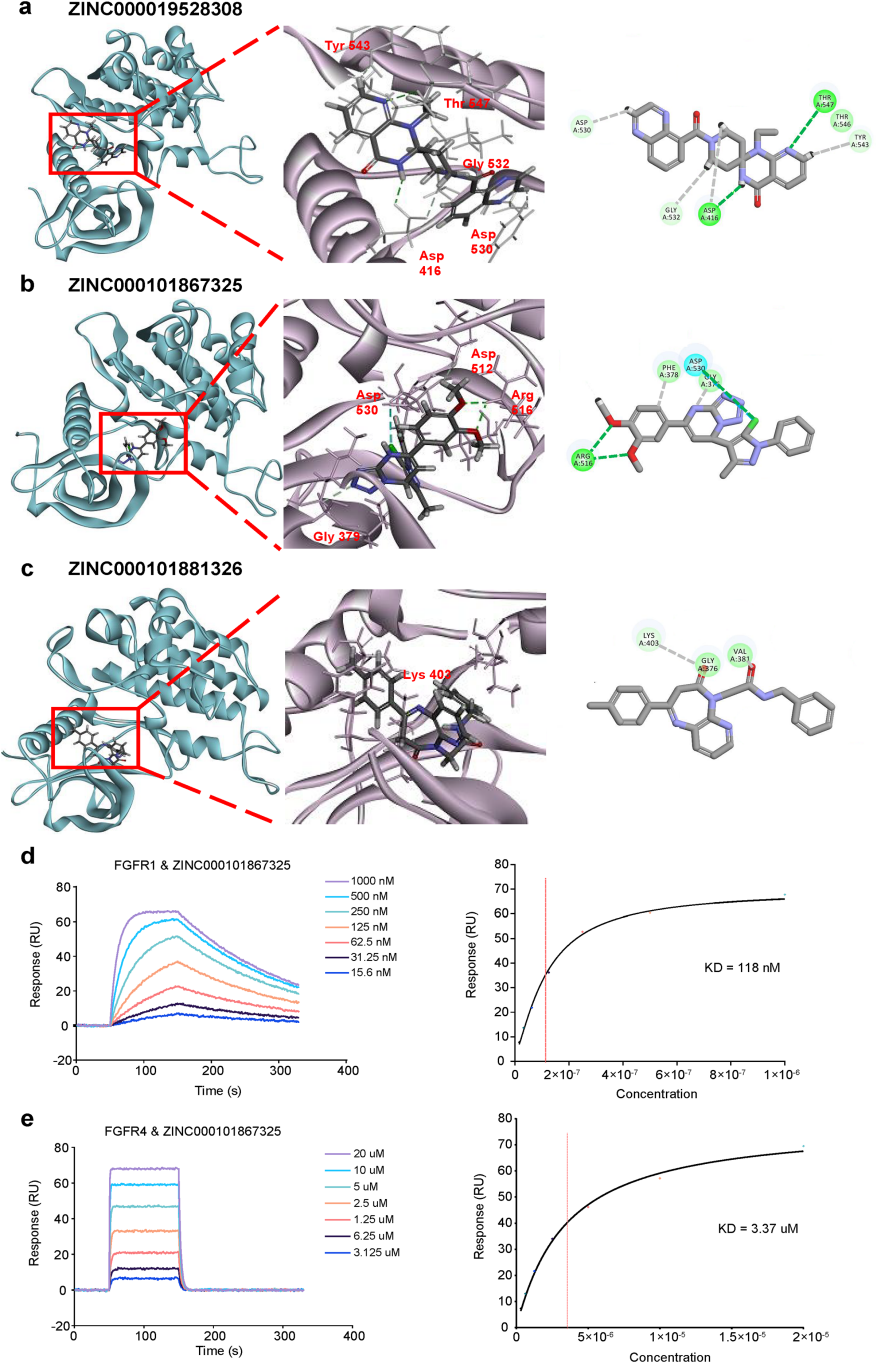
Docked pose of FGFR2 with ZINC000019528308 **(a)**, ZINC000101867325 **(b)**, and ZINC000101881326 **(c)**. **(a-c)** The green dotted line illustrates the hydrogen bond formed between the small molecule compound and the FGFR protein, while the gray dotted line represents the Van der Waals interaction between them. **(d, e)** Validation of the affinity of ZINC000101867325 to the TK domains of FGFR1 **(d)**, FGFR4 **(e)** by SPR. KD refers to the dissociation constant.

Further analysis of the three small molecule compounds based on molecular docking results revealed their binding interactions with the FGFR proteins. Visualization of the three-dimensional structure of the complexes highlighted the potential binding at the hinge regions of FGFRs (**Figures 2a-c**). A two-dimensional bond energy analysis showed that three oxygen atoms in ZINC000101867325 formed hydrogen bonds with Arg516 and Asp530 in the kinase domain’s hinge region. ZINC000019528308 established two hydrogen bonds in the hinge region, while ZINC000101881326 engaged only in Van der Waals interactions with Lys403. Furthermore, kinome selectivity analysis of ZINC000101867325 revealed a propensity for off-target effects on kinase families associated with TK, CMGC, and STE (**Supplementary Figure S4a**). Nonetheless, the binding sites of FGFR2 showed a lower off-target effect comparing to other three FGFR proteins (**Supplementary Figures S4b, c**). To further verify the binding of ZINC000101867325 with FGFRs, we employed Surface Plasmon Resonance (SPR) to assess the affinity of ZINC000101867325 to the TK domains of FGFRs. Because the TK regions of FGFR1–3 are almost identical, so only the TK domains of FGFR1 and FGFR4 were tested here. The results showed that ZINC000101867325 exhibited a strong affinity for the TK domain of FGFR1, but a poor affinity for FGFR4 (**Figures 2d,e**). Accordingly, we tried to test the effect of ZINC000101867325 (Hereinafter referred to as Z325) on the activity of FGFR signaling in cancer cell lines.

### 2.3 Z325 inhibits the activity of FGFR signaling in CRC cell lines

We explored transcriptional alterations in FGFR family genes across normal tissues from the GTEx project (n=9,348) and cancer tissues from the TCGA project (n=9,783) (25, 26). The expression changes in FGFR family genes were evaluated across various cancers. Notably, CRC exhibited the highest cumulative fold changes in these genes (**Supplementary Figure S5a**). Previous cohort studies on CRC have revealed multiple aberrations in FGFR signaling pathways. Consequently, CRC cell lines were chosen to test the effect of Z325 on the activity of FGFR signaling.

**Figure 3:**
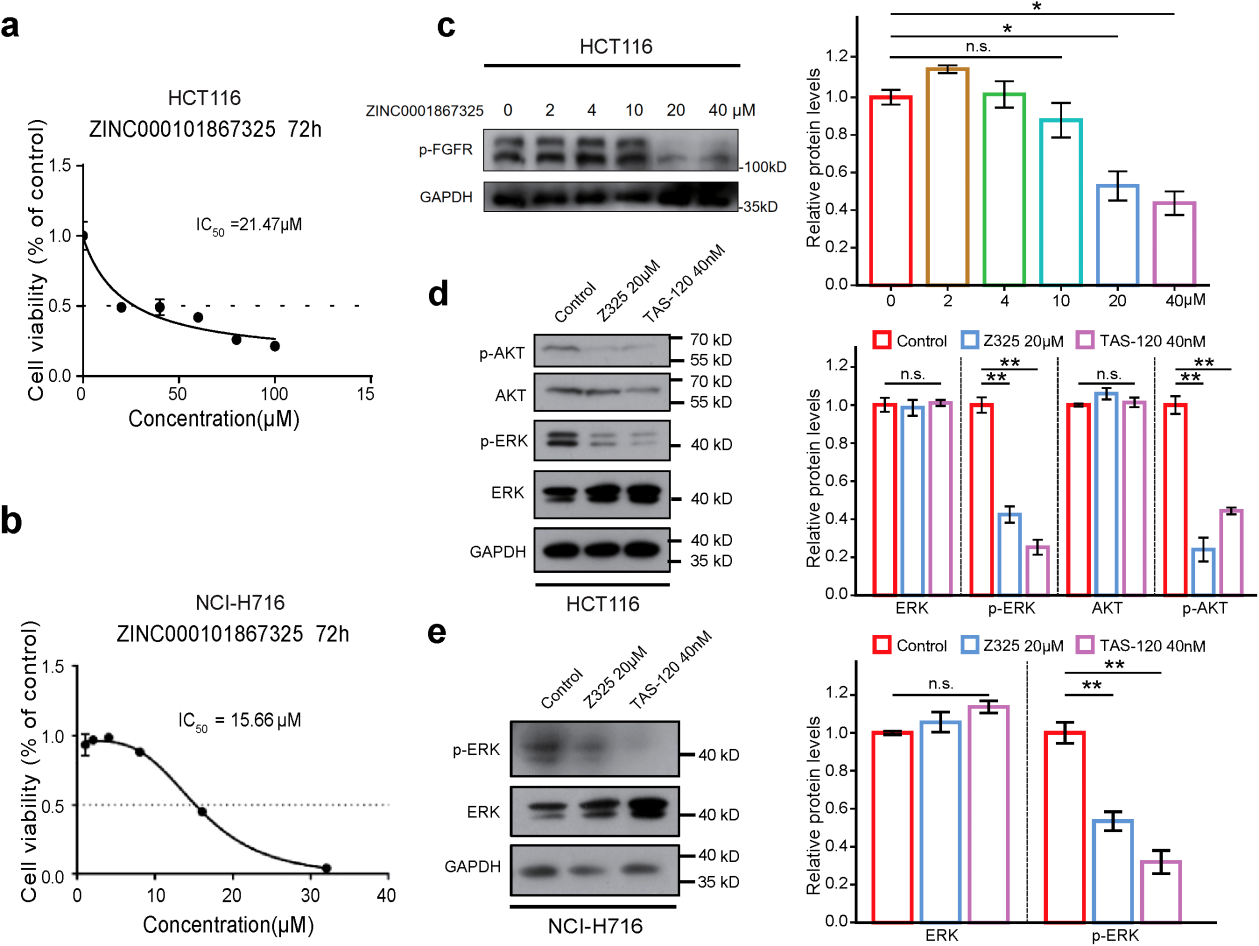
Z325 inhibits the FGFR phosphorylation in CRC cell lines. **(a)** IC_50_ value of ZINC000101867325 measured in HCT116 cell line. **(b)** IC_50_ value of ZINC000101867325 measured in NCI-H716 cell line. **(c)** Effect of ZINC000101867325 on the phosphorylation level of FGFR. **(d)** Effect of ZINC000101867325 on the phosphorylation level of AKT and ERK in HCT116 cell line. **(e)** Effect of ZINC000101867325 on the phosphorylation level of ERK in NCI-H716 cell line. Differences between means were calculated using two-tailed Student’s t-test. *P < 0.05, **P < 0.01.

To determine the optimal dosage of Z325, we initially evaluated its impact on the proliferation of the CRC cell line HCT116 using the CCK8 assay. After 72 hours of treatment with the concentrations of Z325 indicated, a dose-dependent decrease in cell viability was observed, and the IC_50_ for Z325 was determined to be 21.47 μM (**Figure 3a**), and the IC_50_ tested in another CRC cell line NCI-H716 was 15.66 μM (**Figure 3b**), setting the subsequent experimental concentration at 20 μM. Then, we investigated the impact of Z325 on FGFRs phosphorylation. As the dosage increased, the phosphorylation levels of the treatment group progressively diminished, notably at 20 μM (**Figure 3c**; **Supplementary Figure S5b**). Furthermore, the phosphorylation levels of AKT and ERK in the downstream of the FGFR signaling were also inhibited (**Figure 3d**), which was also observed in NCI-H716 (**Figure 3e**), indicating that Z325 effectively inhibits the activation of the FGFR signaling pathway.

### 2.4 Z325 promotes apoptosis in CRC cell lines

**Figure 4:**
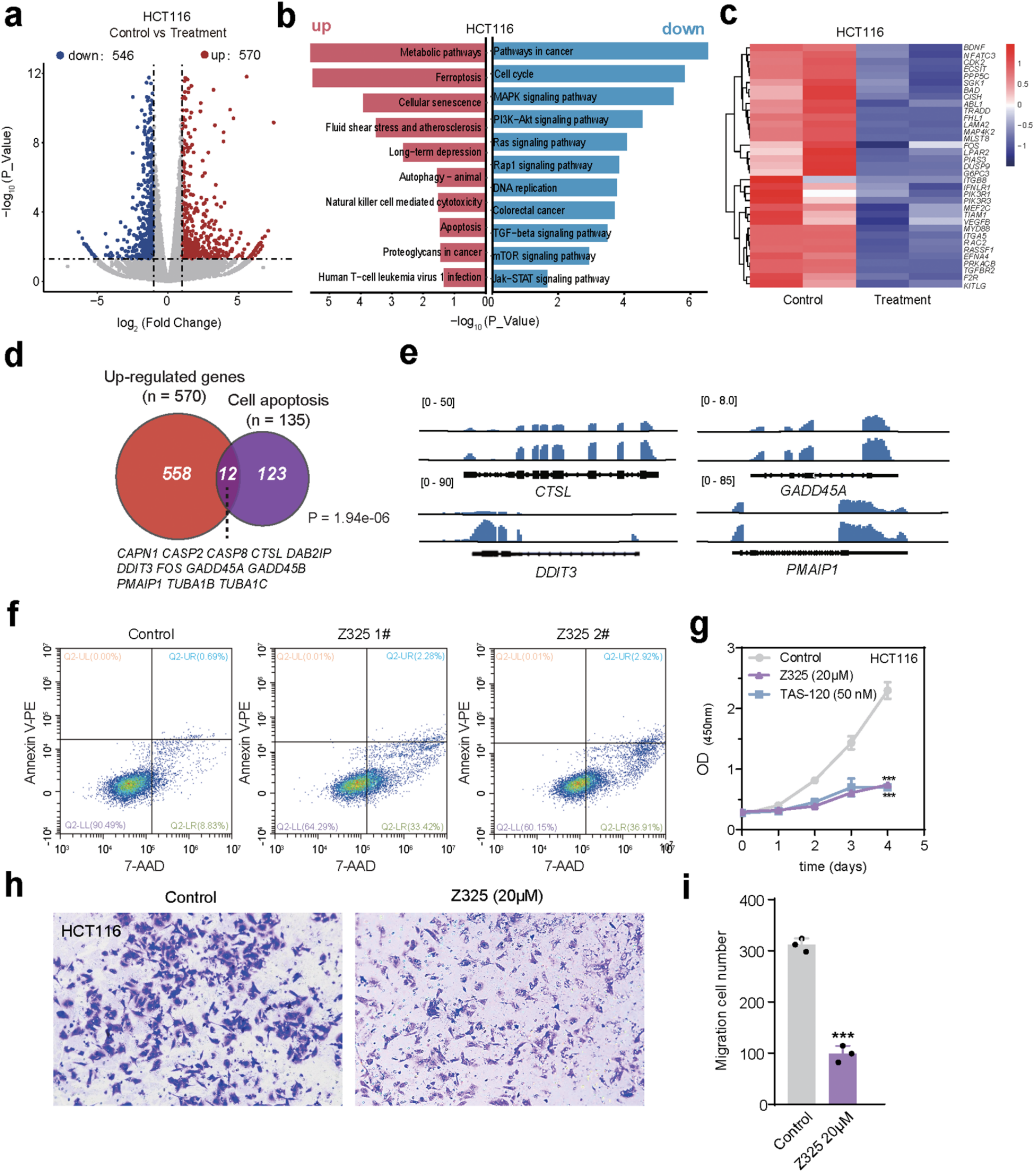
Z325 promotes apoptosis in CRC cell lines. **(a)** Volcano plot displaying the impact of ZINC000101867325 on the transcriptome of HCT116 cell line. |log2FC| >1.5, p <0.05 **(b)** KEGG pathway annotation of differentially expressed genes in the ZINC000101867325-treated HCT116 cell line. Red indicates pathways enriched in up-regulated genes, while blue indicates pathways enriched in down-regulated genes. **(c)** Changes in expression levels of genes related to the FGFR downstream pathway in the control and ZINC000101867325-treated groups. **(d)** Up-regulated genes in the HCT116 cell line treated with Z325 are enriched in cell apoptosis. Cell apoptosis related genes were integrated from KEGG, Reactome and Pathbank. **(e)** Genome browser visualization of representative apoptosis-related genes. **(f)** Detection of cell apoptosis by flow cytometry in HCT116 cell line. Z325 1# and 2# indicates two replicates. **(g)** The proliferation of HCT116 cells decreases as the treatment time with Z325 and TAS-120 treatment. **(h-i)** Transwell experiments of the control group and the Z325-treated group. **(h)** Representative images of cell migration staining; **(i)** Statistics of cell migration numbers. ***P < 0.001.

In the context of tumor pharmacotherapy, the promotion of cancer cell apoptosis is crucial for therapeutic effect (27, 28). Accordingly, we investigated the effect of Z325 on apoptotic cell death of CRC cell lines. We performed RNA-seq on the control and Z325-treated groups, with two biological duplicate repeats. Unsupervised hierarchical clustering (UHC) separated the transcriptomic patterns of the Z325-treated groups from the control groups (**Supplementary Figure S6a**). Differential expression analysis revealed significant up-regulation of 570 genes and down-regulation of 546 genes (**Figure 4a**; |log2FC| >1.5, p <0.05). No difference in the expressions of FGFRs were observed between the control and treated groups (**Supplementary Figure S6b**). KEGG pathway enrichment analysis indicated that up-regulated genes predominantly enhanced pathways related to ferroptosis, apoptosis, and cellular senescence. Conversely, down-regulated genes were significantly involved in cell cycle and cancer pathways, including the MAPK, PI3K-Akt and mTOR signaling pathways (**Figures 4b, c**). We also observed a similar effect of Z325 on NCI-H716 (**Supplementary Figures S6c-e**).

The up-regulated genes in the treated-cells showed significant enrichment in cell apoptosis (**Figure 4d**), such as *CTSL*, a lysosomal cysteine protease crucial for intracellular protein degradation (29); *DDIT3* (CHOP protein), which can inhibit anti-apoptotic protein *BCL-2* expression and enhance pro-apoptotic protein Bax expression (30); *GADD45A*, known to increase mitochondrial membrane permeability and induce apoptosis (31); *PMAIP1*, which by interacting with various anti-apoptotic proteins can activate apoptotic proteins, thereby fostering increased mitochondrial membrane permeability and apoptosis (32) (**Figure 4e**). Furthermore, we employed flow cytometry to assess the effect of Z325 on CRC cell apoptosis. The results showed a significantly higher proportion of early apoptotic cells in the HCT116 cell line treated with 20 uM Z325 for 24 hours, comparing to the control group (**Figure 4f**). And, cell apoptosis happened more severely in NCI-H716 by treatment with 20 uM Z325 (**Supplementary Figures S6f, g**). Meanwhile, we also found that the cell proliferation and migration were decreased in HCT116 and NCI-716 treated with Z325 (**Figures 4g-i**; **Supplementary Figures S6h, i**). In sum, these findings suggest that Z325 could inhibit FGFR signaling pathways, and exhibit anti-CRC activity, highlighting its potential as a small molecule therapeutic agent in tumor treatment.

### 2.5 Prediction of Z325 targeting on FGFR2 mutant

**Figure 5:**
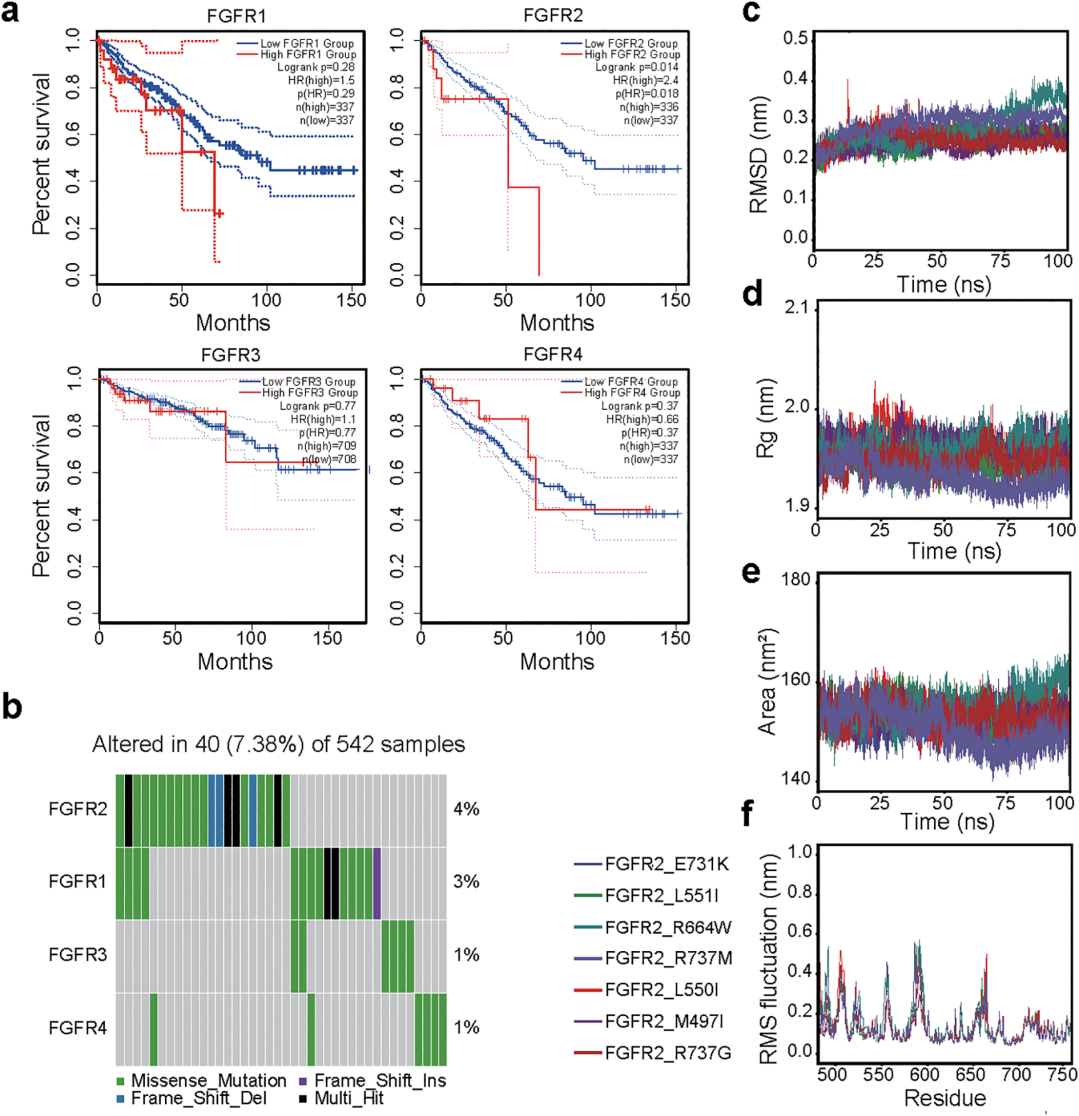
Molecular dynamics simulation of ZINC000101867325 on FGFR mutants. **(a)** Mutation frequency of FGFR1–4 in TCGA colorectal cancer cohort. **(b)** Survival analysis of FGFR low and high expression groups in colorectal cancer patients. **(c-f)** Molecular dynamics simulation of Z325 on FGFR2 with different mutations in root mean square deviation **(c)**, radius of gyration **(d)**, solvent accessible surface area **(e)**, and root mean square fluctuations **(f)**.

Previous studies have demonstrated that genetic variations in FGFRs can enhance the activity of FGFR signaling and promote tumor development (33). However, patients with various FGFR genetic variations exhibit different responses to small molecule inhibitors (8, 34, 35). To further elucidate the anti-CRC activity of Z325 on patients with different FGFRs genetic mutations, we first integrated clinical characteristics to compare survival curves between colorectal cancer patients with low and high expressions of FGFR1-4 (**Figure 5a**). We found that high expression of FGFR2 significantly reduced survival time comparing to FGFR1, FGFR3, and FGFR4. We also analyzed the genetic mutation data of 542 CRC patients from the TCGA project. Among the FGFR family genes, FGFR2 displayed the highest mutation frequency (n=40, 7.38%), predominantly involving missense mutations (**Figure 5b**).

Mutations in the TK domains of FGFRs may alter its activity, to interact with other signaling molecules or regulatory proteins, and promoting tumor development. We identified 7 mutations within the TK domain of FGFR2, showing arginine at position 737 was most frequently mutated to glycine and methionine, followed by glutamic acid at position 731 (**Supplementary Table S1**). Using homology modeling, we constructed the mutated FGFR2 structure and performed molecular docking with Z325 to calculate the binding free energies. The analysis revealed that the mutants of FGFR2 exhibited high binding affinities for Z325.

To further analyze the binding stability of the Z325 and mutated FGFR2, we conducted a 100 ns molecular dynamics simulation (**Figure 5c**). The simulation results indicated that the potential energies of the seven FGFR2 mutants complexed with Z325 were consistently around -680,000 kj/mol. The RMSD for each mutated FGFR2 showed that FGFR2_L550I had significant fluctuations at 15ns before stabilizing, while FGFR2_R664W displayed minor fluctuations, maintaining overall stability throughout the 100ns simulation. The radius of gyration (RG) decreased after initial fluctuations, stabilizing and indicating a tight protein complex (**Figure 5d**). The solvent-accessible surface area (SASA) remained stable across all mutants except for a slight decrease in FGFR2_R664W (**Figure 5e**). The RMSF for each amino acid was under 0.6 Å, demonstrating the overall structural stability of the protein-small molecule complexes (**Figures 5f**). Overall, our results indicate that the FGFR2 mutants form a relatively stable complex with Z325, indicating it as a potential small molecule inhibitor for patients with mutated FGFR2 CRC.

## 3 Discussion

In this study, we identified three potential small molecule inhibitors that target the TK domains of FGFR proteins through extensive virtual screening. Compared to the known FGFR inhibitor TAS-120, these novel molecules display comparable efficacy in molecular dynamics simulation, underscoring the potential of structure-based diversification in large molecular libraries for the discovery of novel small molecule inhibitors.

We specifically selected Z325 for further validation, based on the bond types and stability metrics of the docking poses of FGFR proteins. CRC is a devastating disease with limited treatment options. Here, we suggest that Z325 might be a promising therapeutic agent for CRC patients with FGFR mutations. This compound could significantly inhibit the FGFR signaling pathway and induce tumor cell apoptosis in CRC cell lines, in line with the previous studies, showing that inhibition of the FGF/FGFR pathway with small molecule inhibitors, such as NSC12 or erdafitinib, can trigger apoptosis in human squamous cell carcinoma cells (36, 37). Further study will perform FGFR knockdown and construct FGFR-mutant models in suitable CRC cell lines to establish FGFR dependency and evaluate potential off-target effects, and focus on the examination of Z325 on normal healthy cell lines and other CRC cell lines to more comprehensively assess its toxicity, and examining its impact on apoptosis and cell proliferation. Additionally, it is also necessary to further optimize the molecular structure of Z325. *In vivo*, we expect to use the optimized Z325 in tumor xenograft models to evaluate the tolerability of this small molecule in inducing tumor stagnation and regression. It is anticipated that CRC patients may also achieve similar therapeutic benefits at effective doses in future clinical trials.

Enhanced FGFR signaling plays a crucial role in the activation of key survival and proliferation pathways among multiple cancer types, by genetic alterations such as amplifications, mutations, or chromosomal translocations. These alterations are involved in tumor development, progression, and resistance to anti-cancer therapies. Specifically, targeting a common FGFR2 missense mutation in CRC, molecular docking simulations suggested that Z325 also inhibits mutant FGFR2. However, it is important to note that the effectiveness of FGFR inhibitors may be limited by intra-tumor heterogeneity. For instance, early trials with selective TK inhibitors showed promising results in patients with FGFR fusions and certain FGFR2 amplifications, but were less effective against other FGFR alterations (38, 39).

In conclusion, Z325 shows promise as an inhibitor for FGFR proteins. However, future studies are required to optimize its molecular structure, and elucidate its inhibitory effects on various FGFR aberrations through *in vivo* and *in vitro* studies. The differential inhibitory effects of Z325 on various FGFR aberrations could influence the frequency and severity of its side effects.

## 4 Materials and methods

### 4.1 Molecular docking

To perform molecular docking, we utilizing High Performance Computing system (HPC) from Oujiang Laboratory (Zhejiang Lab for Regenerative Medicine, Vision, and Brain Health) through parallel computing by setting 200 standard CPU cores and corresponding sufficient memory in Slurm script, and computing resources are dynamically adjusted according to actual needs.

The three-dimensional structure of the FGFR1/2/3/4 protein and the amino acid positions within the tyrosine kinase domain were downloaded from the PDB database (40). The predicted three-dimensional structure of FGFR proteins were obtained from AlphaFold2 (41). All non-kinase regions were removed from the protein’s PDB file using Chimera. AutoDock Vina was employed to identify any small molecules, such as water, and to append polar hydrogen atoms to the receptor protein (23). All atoms within a 5.0Å spatial distance in the ATP-binding region were extracted based on atomic distances around amino acid residues, and the coordinates for the docking box size were established.

Small molecule data were extracted from the ZINC15 database, with approximately 2.8 million commercially available (42), lead-like drug molecules being screened. Initially, mol2 format compressed files containing all small molecules were downloaded and decompressed to obtain the 3D conformation mol2 files. These files were then converted to PDB format using Open Babel. AutoDock Tools was used to add hydrogen atoms to the cleaned receptor files and convert the crystal structure of small molecule compounds into pdbqt file format.

The protein structure obtained was compared with the structure of known protein-bound small molecules. A docking box size was set based on the extension of known small molecules within a 5 Å range of surrounding amino acids. The docking box parameters are saved into a script file recognizable by AutoDock Vina, ensuring that the software can accurately execute the docking calculations. Each potential ligand-receptor conformation is evaluated based on its exclusion volume and binding free energy. To enhance docking efficiency and minimize computational redundancy, the number of docking poses is limited to nine. The docking results are then ordered by free energy, from lowest to highest, to facilitate the identification and screening of the most promising ligand-receptor binding modes. For each small molecule compound, the conformation with the lowest binding free energy is extracted, along with its corresponding docking free energy. Finally, compounds with the lowest free energy across all docking results are selected for further analysis, focusing on their binding interactions with the target protein to assess their potential as inhibitors.

Gromacs2020.5 was utilized to perform a 100ns molecular dynamics (MD) simulation and identify potential binders to FGFR1–4 on three small molecule compounds and the known inhibitor TAS-120. The protein systems were constructed using the force-field position for semi-flexible docking programs. Multiwfn software processed the structure of the target small molecule compounds to calculate the RESP atomic charges, and Sobtop was used to generate the topology file for ordinary organic molecules. The GAFF standard was used to construct the topology and gro files for the small molecule compounds. Each ligand complex was placed in a dodecahedral model box and solvated with water molecules. A specific gap of 1Å was maintained between the protein periphery and the box edge, and the system’s charge was balanced by adding sodium or chloride ions. The energy of the system was minimized using both the conjugate gradient and steepest descent methods, followed by several steps of slow heating and setting of solute molecules limiting conditions. The biological system was stabilized in an isothermal and isobaric environment, the simulation time was set to 100 ns, and other settings were left as default. Upon completion of the simulation, the molecular dynamics simulation trajectory was extracted.

### 4.2 Cell culture

Human colorectal cancer cell lines HCT116 and NCI-H716 were presented by Professor Zhixiong Dong from Wenzhou Medical University. The HCT116 were cultured in Dulbecco’s Modified Eagle Medium (DMEM) enriched with 10% fetal bovine serum (FBS), 1% L-glutamine, and 1% penicillin-streptomycin. The NCI-H716 were cultured in 1640 Medium enriched with 10% FBS and 1% penicillin-streptomycin. Cultures were maintained in a cell incubator at 37°C with a 5% CO_2_ atmosphere.

### 4.3 Kinome selectivity analysis

KinomeFEATURE was employed to conduct a comprehensive kinome selectivity analysis on small molecule compounds (43). The analysis focused on the FGFR binding sites of each compound, comparing the sequence similarity of these sites with those present in the kinome database. It is important to note that a lower Pocket Feature Score indicates a higher likelihood of off-target effects. To further elucidate potential off-target kinases, the online tool kinmap was utilized to visualize the kinase phylogenetic tree (44).

### 4.4 Pan-cancer evaluation of expression changes in FGFR family genes

The pan-cancer public transcriptome data resource was utilized to investigate transcriptional changes in FGFR family genes across normal and cancerous tissues. Transcriptome data from normal tissues were derived from the GTEx project (n=9,348), while data for cancerous tissues were derived from the TCGA project (n=9,783). Expression data from various sources and batches were standardized and adjusted using the UCSC Toli process. For each cancer type, fold change metrics were employed to quantify the differences in gene expression between normal and cancerous tissues. The cancer types were then ranked based on the cumulative fold changes observed in the FGFR family genes.

### 4.5 RNA sequencing

RNA-seq libraries were prepared as previously described (45). Briefly, cells were amplified to obtain cDNA by the SMART-Seq2 protocol (N712; Vazyme, Nanjing, China). cDNA was quantified by Qubit 3.0 (Thermo Fisher) and 5 ng cDNA was used for DNA library construction with TruePrep DNA Library Prep Kit V2 for Illumina (TD502, Vazyme) following the manufacturer’s instructions. The libraries were sequenced on an Illumina Novaseq 6000 platform and 150 bp paired-end reads were generated.

### 4.6 RNA-seq data processing

For RNA-seq raw reads, low-quality bases and adapter sequences were trimmed using TrimGalore v0.4.5. The clean reads were then aligned to the human reference genome GRCh38 using STAR v2.6.1c (46). The expression levels for each gene were quantified as Fragments Per Kilobase of transcript per Million mapped reads (FPKM) using RSEM v1.3.1 (47). Differentially expressed genes between the control and ZINC000101867325-treated groups were identified using a two-tailed t-test. KEGG pathway enrichment analysis was conducted using the KOBAS online tool (http://bioinfo.org/kobas) (48). RNA-seq tracks were visualized using the IGV Genome Browser (49).

### 4.7 Survival analysis

The survival data for colorectal cancer patients were sourced from the GDC Portal database. Patients were categorized into either a low or high expression group based on the median gene expression values of FGFR family genes. We conducted survival analyses using the Kaplan-Meier method and the log-rank test. All statistical analyses were carried out using R software (version 3.4.4) along with the following packages: “samr,” “ggpubr,” “survival,” “survminer,” “timeROC,” and “ROCit.”

### 4.8 Western blot

When the confluence degree of the 24-well plate reached more than 80%, the samples were collected and sonicated for quantification by BCA. The cells were fully lysed and mixed with 5×SDS loading Buffer at the concentration of 1ug/uL, and then denatured at 100°C for 5min in a metal bath, followed by 8% SDS-PAGE gel electrophoresis. The PVDF membrane was preactivated with 100% methanol, and proteins were transferred from the gel to a 0.2µm PVDF membrane by wet transfer. The membrane was then blocked in 5% skim milk containing sodium azide for 1h at room temperature on a shaker. After one wash with 1×TBST, the membranes in which the target bands were located were cut off separately and incubated with 1×TBST diluted primary antibody at 4°C overnight on a shaker. Before incubation with secondary antibodies, the cells were washed three times with a 1×TBST shaker for 5min each. Secondary antibodies diluted in 1×TBST were incubated for 1h at room temperature on a shaker. They were washed 3 times with a 1×TBST shaker for 5min each time. After washing, the bands were incubated with Pierce™ ECL Western Blotting Substrate (Thermo, PI32209) for 1min before visualization and exposure. The antibodies to be used in this study were: anti-phospho-FGFR (Cell Signaling Technology, cat. #3471), anti-GAPDH (Cell Signaling Technology, cat. #2118).

### 4.9 Flow cytometry

Cells were collected according to the specified treatment time, processed into single cell suspension with a 40-mesh cell screen, and the number of cells in each group was guaranteed to be no less than 1×10^5^ cells, and three biological replicates were set. Used Annexin V-PE/7-AAD apoptosis detection kit (KeyGEN, KGA1016) detected apoptosis. According to the manufacturer’s instructions, after the cell suspension was centrifuged to dispose of the supernatant, 5 × Binding Buffer was diluted into 1× working solution with enzyme-removed water, 500μL pre-cooled working solution was added to the cells, and 5 μL Annexin V and 10μL 7-AAD were added to the double staining tube, respectively. Single staining tubes were added with 5 μL Annexin V or 10 μL 7-AAD, respectively. The samples were evenly divided into two parts, one part was left untreated temporarily, and the other was bathed with metal at 100°C for 5 min to cause cell death. When cooled to room temperature, the two tubes were mixed and 1× Binding Buffer was added to 1.5 mL and evenly divided into three parts. The blank tubes were not treated, and all samples were incubated at room temperature in the dark for 5 min and cell apoptosis was checked by Ctyo II flow cytometry and analyzed using FlowJo software.

### 4.10 Small molecule inhibitors

All small molecule inhibitors were screened from ZINC-15 database, and the small molecule compounds were synthesized and provided by TOPSCIENCE (Shanghai, China). ZINC000101867325 was dissolved in DMSO.

### 4.11 IC_50_

The cells were seeded in 96-well plates with five duplicate Wells in each group, and 100 μL DPBS was added to the Wells around the sample Wells to prevent liquid evaporation. After the cells adhered to the wall, different concentrations of drug-treated medium were replaced, and set the blank control groups. IC_50_ assay was performed using the CCK8 kit (Dojindo, Kyushu, Japan), and cell proliferation was determined at the indicated times according to the manufacturer’s protocol. Cell viability was determined by measuring the absorbance at 450 nm. The formula was calculated as follows: cell viability (%) = [A (treat) -A (blank)]/[A (control) -A (blank)] ×100.

### 4.12 SPR

SPR measurements were performed on the Biacore 8K instrument (GE Healthcare, Piscataway, NJ, USA). FGFR1 and FGFR4 protein TK domains (20 μg/mL, pH 4.5) were immobilized on Series S sensor chips (GE Healthcare, Piscataway, NJ, USA) according to the standard amine coupling procedure (approximately 15,000 RU). PBS (BR100672, pH 7.2–7.4, Cytiva) with 1% DMSO was used as the immobilization buffer. After immobilization, the Z325 solution was prepared by diluting the stock solution with running buffer. Solutions of Z325 at various concentrations were then injected simultaneously at a flow rate of 20 μL/min for an association phase of 100 seconds at 25°C. The final curves were obtained by subtracting blank sensorgrams. Experimental data were collected using Biacore 8K Manager software (GE Healthcare, Piscataway, NJ, USA) and analyzed by fitting to an appropriate binding model to obtain the equilibrium dissociation constant (KD).

### 4.13 Transwell

Cells were seeded in 6-well plates at 1×10^6 cells/well per well and the chambers were washed. Cells were starved for 12 h to eliminate the influence of serum. The transwell chambers were equilibrated with DMEM basal medium for 30 minutes, with 500 μL in the lower chamber and 200 μL in the upper chamber. After chamber equilibration, 500 μL DMEM complete medium containing 20% FBS was added to the lower chamber. Cells in the 6-well plates were digested with 0.25% trypsin, resuspended in 1 mL DMEM, centrifuged at 1000 rpm for 3 minutes, and the cells were resuspended and counted. Cells were seeded into the upper chambers of 24-well plates at a density of 1×10^5 cells/well in a final volume of 200 μL serum-free medium per well. After placing in an incubator static for 5 minutes, the setup was observed under a microscope and photographed, then placed in a 37°C, 5% CO2 incubator for static culture for 24 hours. The upper chambers were rinsed with PBS, fixed with 4% PFA for 20 minutes with 500 μL in the lower chamber and 200 μL in the upper chamber. After 20 minutes, the chambers were inverted and dried on the lid of the 24-well plate. The upper chambers were rinsed with PBS and stained with crystal violet in the dark for 30 minutes, with 500 μL in the lower chamber and 200 μL in the upper chamber. The upper chambers were rinsed with PBS and dried by inverting on paper for a few seconds. Non-migrated cells were removed with a dry cotton swab, and the chambers were placed in a dry 24-well plate for observation under a microscope. Five images were captured at 100× magnification, and cell counting and analysis were conducted using Image J.

## Data Availability

The datasets presented in this study can be found in online repositories. The names of the repository/repositories and accession number(s) can be found in the article/**Supplementary Material**. Publicly available datasets analyzed in this work are available in GEO. All sequencing data of cell lines generated in this study have been deposited in GEO under accession GSE267868.
