# Supplementary material for: Identification of small molecule inhibitors targeting FGFR through molecular docking-based screening
Source: Front Oncol. 2026 Jan 29;16:1733391. doi: 10.3389/fonc.2026.1733391 (PMC12893941; doi:10.3389/fonc.2026.1733391)
Supplement: Supplementary file 1 [file DataSheet1.pdf]

Supplementary information files

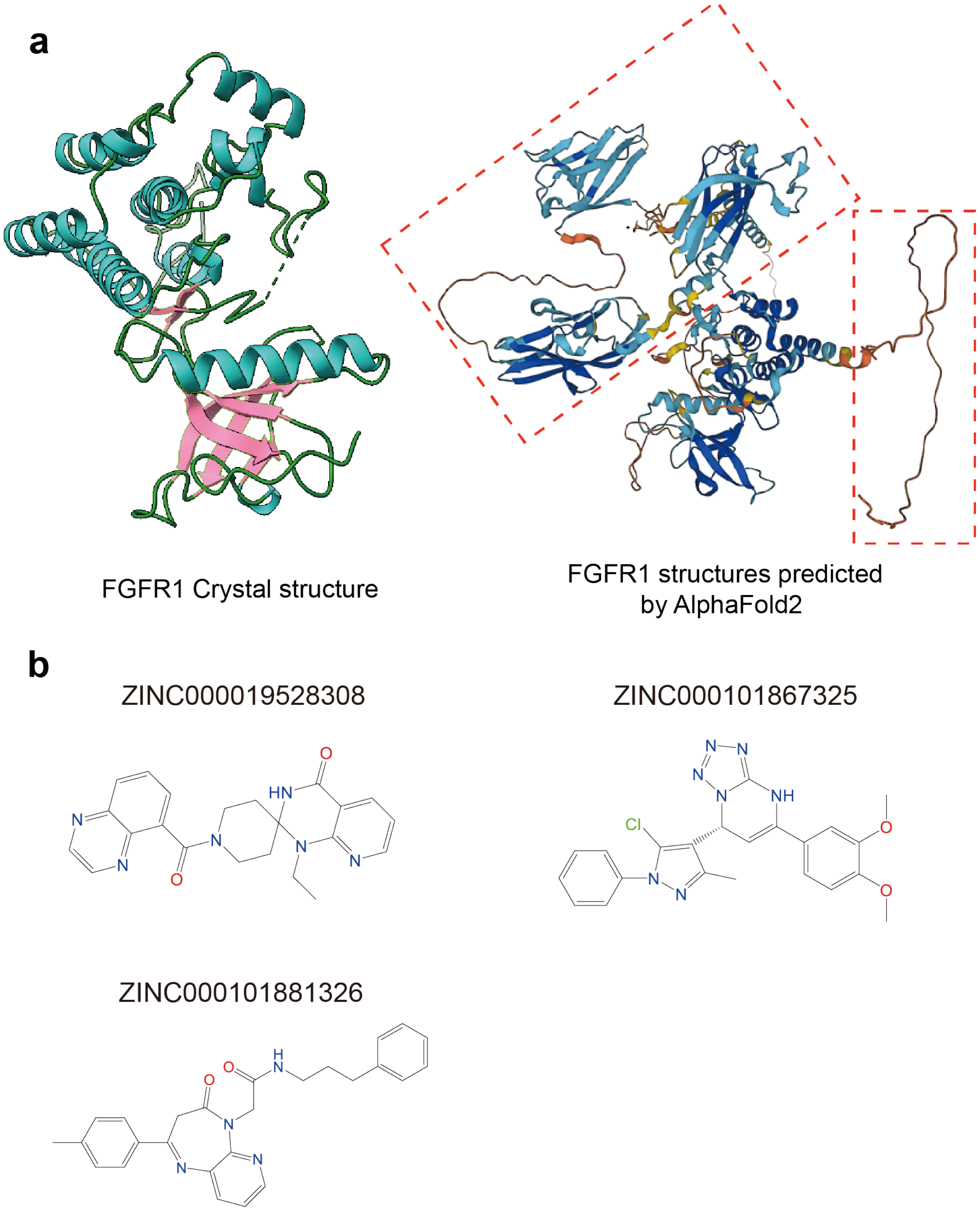

Figure S1. The structure of FGFR1 from PDB and AlphaFold2.

(a) The diagram displays PDB data acquisition results for FGFR1 on the left, with AlphaFold prediction results on the right. The section enclosed in a box highlights the flexible residues within the FGFR1 protein. **(b)** The structures of three potential FGFRs inhibitors.

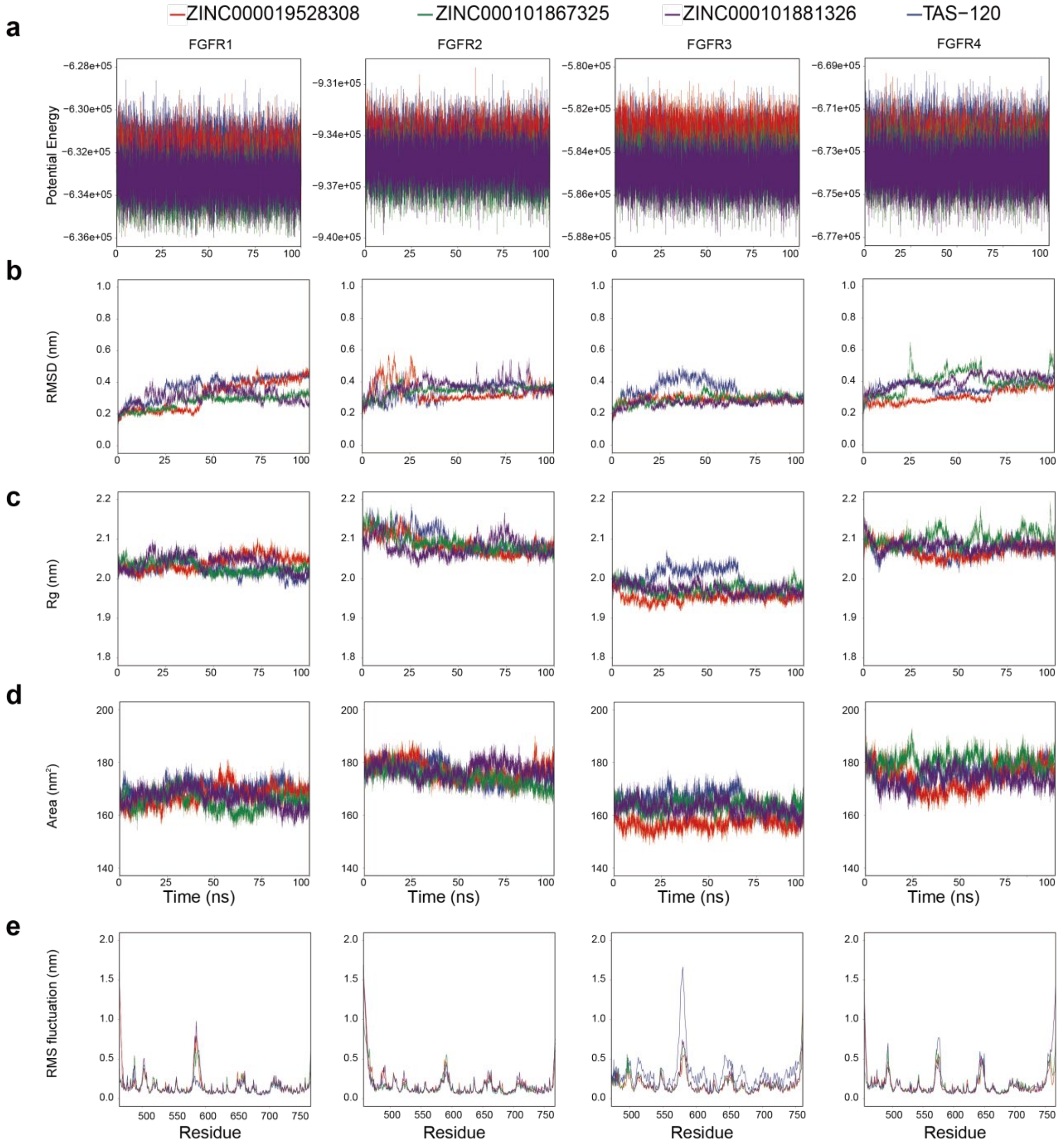

Figure S2 Characterization of potential small molecules targeting FGFRs.

(a-e) Molecular dynamics simulation of three small molecule compounds (ZINC000019528308, ZINC000101867325, ZINC000101881326) and the known FGFR inhibitor TAS-120 in potential energy **(a)**, root mean square deviation **(b)**, radius of gyration **(c)**, solvent accessible surface area **(d)**, and root mean square fluctuations **(e)**.

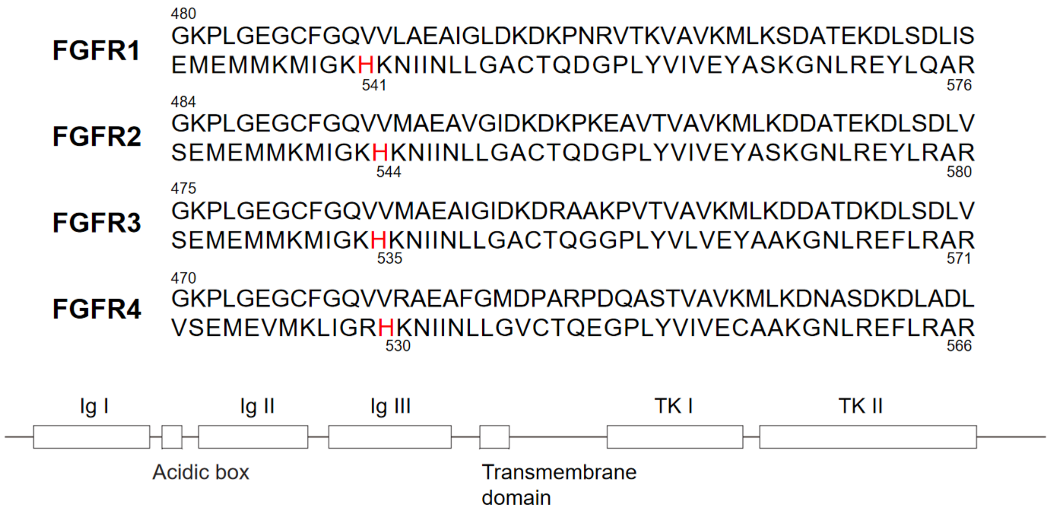

Figure S3. Structure of tyrosine kinase sub-domains in FGFRs.

FGFRs consist of conserved domains including three immunoglobulin (Ig)-like domains (Ig I, Ig II and Ig III), a transmembrane domain and two tyrosine kinase (TK) sub-domains. ZINC000019528308, ZINC000101867325, and ZINC000101881326 bind to the TK I domains of FGFRs (from ~470 to ~580 AA). Histidine (H) is highlighted in red.

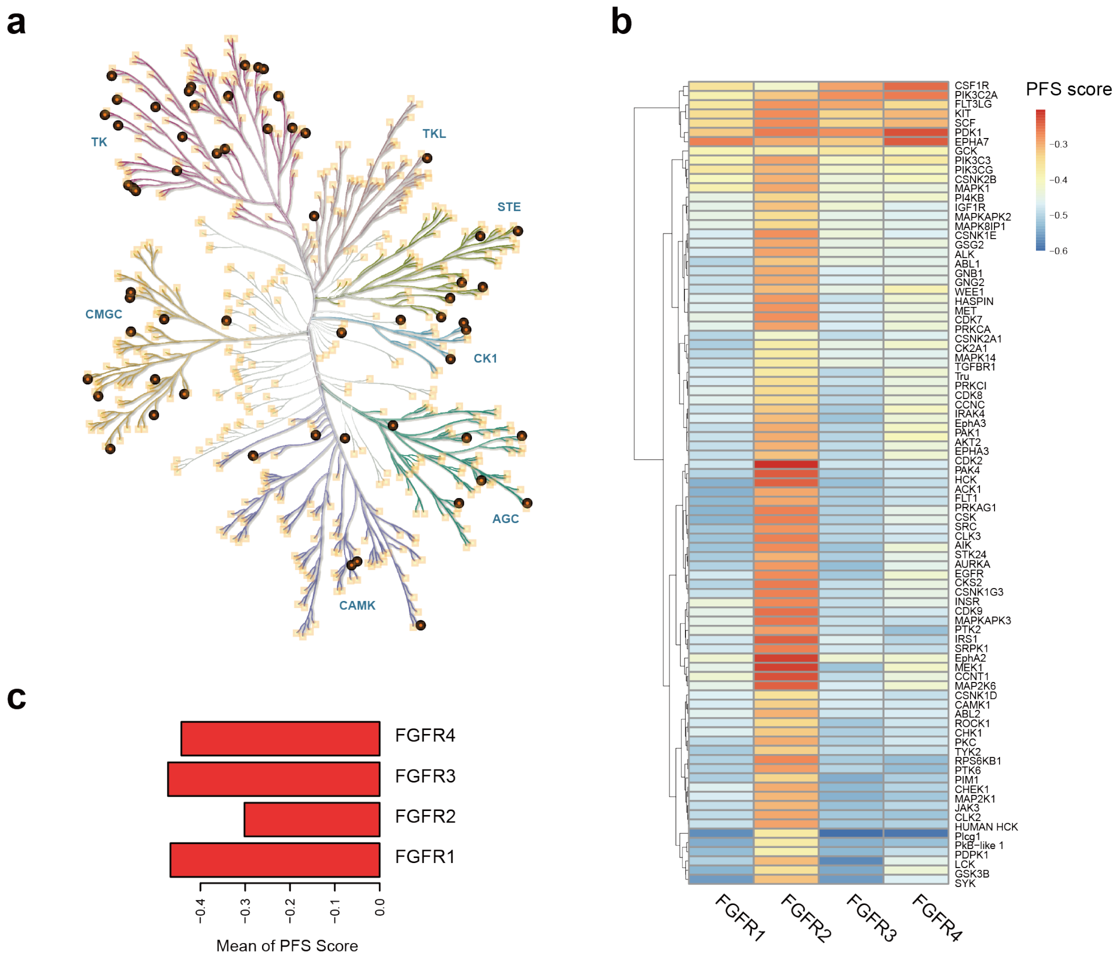

Figure S4 Kinome selectivity analysis of ZINC000101867325.

(a) Kinase tree illustration of the kinases that may be susceptible to off-target effects from FGFR proteins. **(b)** The Pocket Feature Score (PFS) associated with the four FGFR protein binding sites. A lower PFS indicates a higher likelihood of off-target effects. **(c)** The mean PFS score for these four FGFR proteins binding sites.

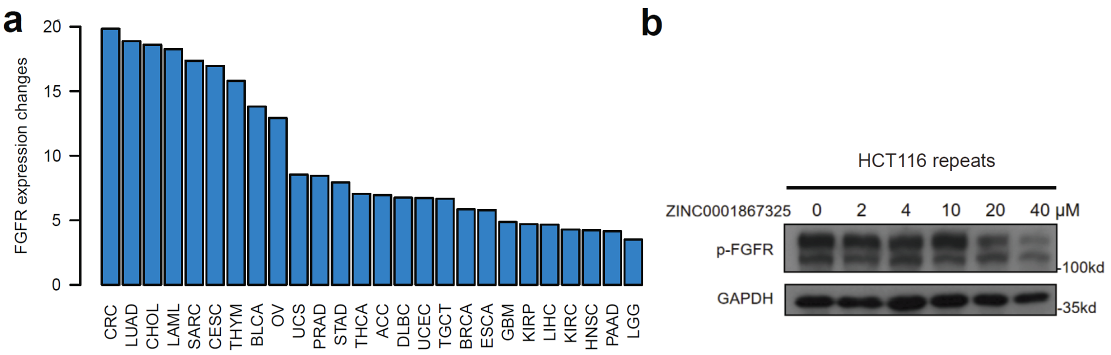

Figure S5. Z325 inhibits the FGFR phosphorylation in CRC cell lines.

(**a**) Pan-cancer analysis of transcriptional changes of FGFR family genes. **(b)** Effect of ZINC000101867325 on the phosphorylation level of FGFR (Repeats).

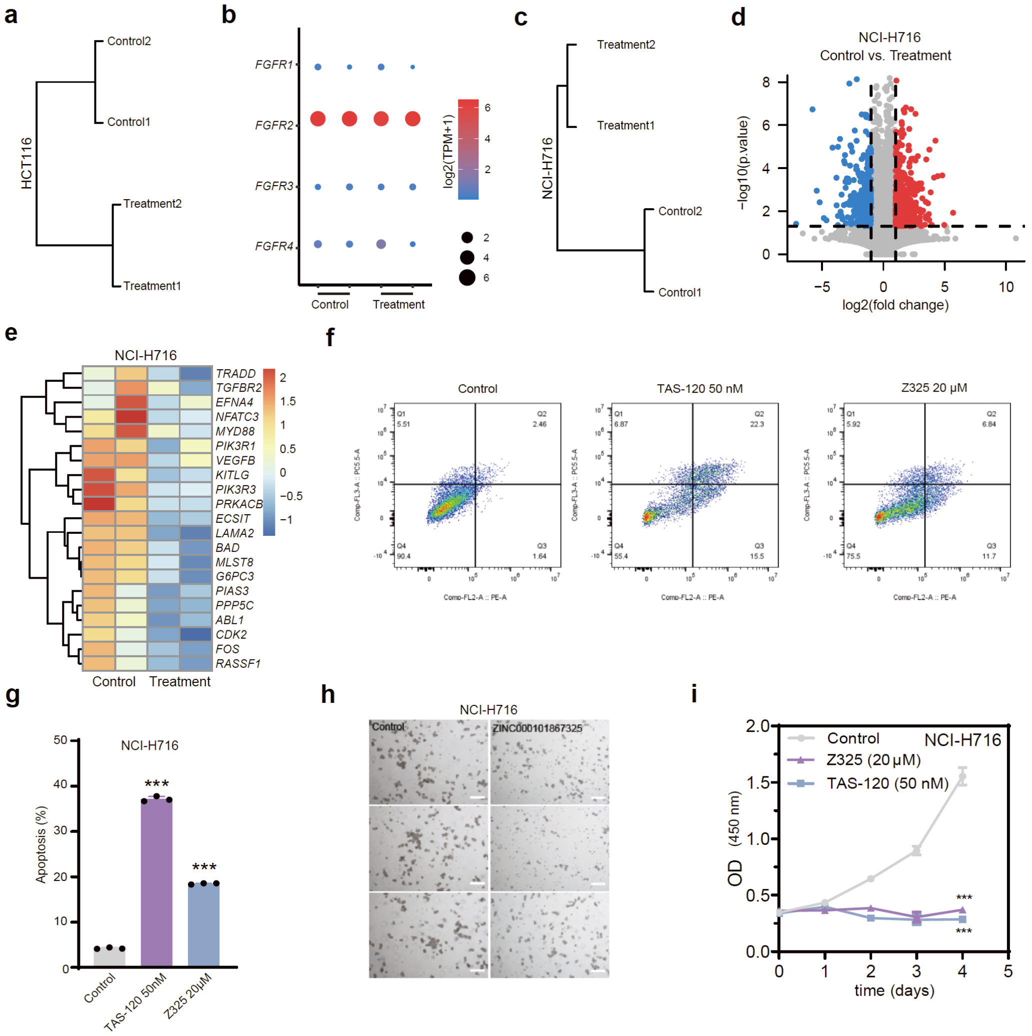

Figure S6 Z325 promotes apoptosis in NCI-H716 cell lines

(a) Unsupervised hierarchical clustering of gene expressions in the control and Z325-treated groups from RNA-seq of two biological duplicates in HCT116. **(b)** Expression level of FGFRs in control and ZINC000101867325-treated groups. **(c)** Unsupervised hierarchical clustering of gene expressions in the control and Z325-treated groups from RNA-seq of two biological duplicates in NCI-H716. **(d)** Volcano plot displaying the impact of ZINC000101867325 on the transcriptome of NCI-H716 cell line. |log2FC| >1, $p < 0.05$. **(e)** Changes in expression levels of genes related to the FGFR downstream pathway in the control and ZINC000101867325-treated groups. **(f and g)** Cell apoptosis in NCI-H716 after treatment with TAS-120 and ZINC000101867325. *** $p < 0.001$. **(h)** Representative images of NCI-H716 cell line in control and treatment groups. **(i)** Cell proliferation in NCI-H716 after treatment with TAS-120 and ZINC000101867325. *** $p < 0.001$.
