# Supplementary material for: Identification of small molecule inhibitors targeting FGFR through molecular docking-based screening
Source: Front Oncol. 2026 Jan 29;16:1733391. doi: 10.3389/fonc.2026.1733391 (PMC12893941; doi:10.3389/fonc.2026.1733391)
Supplement: Supplementary file 2 [file DataSheet2.docx]

The relevant parameters and configuration files of molecular docking used in this paper are described in detail as follows:

Utilizing AutoDock Vina, we first verified the absence of any extraneous small molecules, such as water, within the protein file. Subsequently, we appended polar hydrogen atoms to the receptor protein and archived the refined structure in PDB format. We then procured the relevant PDB files depicting the binding conformation of FGFR1-4 and their associated small molecule compounds from the PDB database. Employing PyMOL, we initially computed the RMSD between the protein structure derived from AlphaFold and the one sourced from the PDB database, ensuring the RMSD value remained below 1Å. Subsequently, we utilized a script designed to ascertain atomic distances surrounding amino acid residues to extract all atoms within a 5.0Å radius in the ATP binding domain, thereby determining the coordinates for the docking position box dimensions. The coordinates for FGFR1/2/3/4 are as follows: 1) center_x: -21.5, center_y: -2.5, center_z: 3.4, size_x: 32.7, size_y: 39.2, size_z: 47.5; 2) center_x: -17.0, center_y: -5.7, center_z: 6.9, size_x: 48.9, size_y: 41.4, size_z: 38.9; 3) center_x: -16.1, center_y: 5.6, center_z: 8.4, size_x: 50.7, size_y: 34.7, size_z: 39.8; 4) center_x: -113.4, center_y: -10.5, center_z: 6.5, size_x: 50.3, size_y: 48.8, size_z: 43.5. The resolution was set at 0.375Å.

For the processing of small molecule compounds, data were extracted from the ZINC15 database, yielding approximately 2.8 million purchasable small molecules based on lead-like drug criteria. Initially, we downloaded and decompressed the mol2 format archives to obtain the 3D conformation mol2 files of the small molecule compounds. We then employed Open Babel to transform the file format to PDB and subsequently utilized AutoDock Tools to integrate polar hydrogen into the cleaned receptor file, converting it, along with the crystal structure of the small molecule compound, into the pdbqt file format.

The molecules mol2 files are listed as follows:

**ZINC000019528308.mol2**

@<TRIPOS>MOLECULE

ZIN1

82 85 1

SMALL

USER_CHARGES

@<TRIPOS>ATOM

1 C1 -17.444 9.578 7.377 C.3 1 ZIN1 0.048

2 C2 -16.717 8.288 7.656 C.3 1 ZIN1 -0.062

3 C3 -16.687 7.779 8.943 C.3 1 ZIN1 0.007

4 C4 -16.023 6.599 9.206 C.3 1 ZIN1 0.014

5 C5 -15.381 5.916 8.169 C.3 1 ZIN1 -0.011

6 C6 -14.671 4.655 8.441 C.3 1 ZIN1 0.054

7 N7 -14.629 4.208 9.645 N.3 1 ZIN1 -0.248

8 C8 -14.046 3.052 10.048 C.3 1 ZIN1 0.103

9 C9 -14.744 2.278 10.993 C.3 1 ZIN1 0.037

10 C10 -14.184 1.095 11.435 C.3 1 ZIN1 0.022

11 C11 -12.953 0.708 10.937 C.3 1 ZIN1 0.119

12 N12 -12.318 1.444 10.052 N.3 1 ZIN1 -0.241

13 C13 -12.812 2.593 9.587 C.3 1 ZIN1 0.161

14 N14 -12.048 3.264 8.649 N.3 1 ZIN1 -0.291

15 C15 -10.599 3.308 8.861 C.3 1 ZIN1 0.236

16 C16 -10.252 4.498 9.718 C.3 1 ZIN1 0.235

17 O17 -9.950 4.339 10.883 O.3 1 ZIN1 -0.272

18 N18 -10.279 5.738 9.193 N.3 1 ZIN1 -0.360

19 C19 -9.942 6.896 10.026 C.3 1 ZIN1 0.123

20 C20 -9.532 8.067 9.131 C.3 1 ZIN1 0.024

21 C21 -9.915 9.384 9.808 C.3 1 ZIN1 0.040

22 C22 -9.552 10.536 8.907 C.3 1 ZIN1 -0.058

23 C23 -9.236 10.302 7.581 C.3 1 ZIN1 0.007

24 C24 -8.902 11.359 6.754 C.3 1 ZIN1 0.001

25 C25 -8.886 12.648 7.252 C.3 1 ZIN1 0.000

26 C26 -9.203 12.881 8.577 C.3 1 ZIN1 0.001

27 C27 -9.541 11.825 9.404 C.3 1 ZIN1 0.007

28 C28 -12.530 3.875 7.567 C.3 1 ZIN1 0.231

29 O29 -11.766 4.399 6.783 O.3 1 ZIN1 -0.273

30 C30 -14.018 3.919 7.301 C.3 1 ZIN1 0.163

31 C31 -15.417 6.436 6.872 C.3 1 ZIN1 0.014

32 C32 -16.088 7.614 6.623 C.3 1 ZIN1 0.007

33 H01 -18.387 9.589 7.924 H 1 ZIN1 0.000

34 H02 -16.829 10.419 7.698 H 1 ZIN1 0.000

35 H03 -17.643 9.660 6.309 H 1 ZIN1 0.000

36 H04 -15.687 8.030 7.901 H 1 ZIN1 0.000

37 H05 -17.730 7.525 9.134 H 1 ZIN1 0.000

38 H06 -16.232 8.543 9.573 H 1 ZIN1 0.000

39 H07 -16.823 5.919 9.498 H 1 ZIN1 0.000

40 H08 -15.263 6.824 9.954 H 1 ZIN1 0.000

41 H09 -14.791 5.040 8.439 H 1 ZIN1 0.000

42 H10 -14.001 3.820 8.643 H 1 ZIN1 0.000

43 H11 -14.176 4.934 10.181 H 1 ZIN1 0.000

44 H12 -13.362 3.492 9.322 H 1 ZIN1 0.000

45 H13 -15.639 1.959 10.459 H 1 ZIN1 0.000

46 H14 -14.898 2.906 11.870 H 1 ZIN1 0.000

47 H15 -14.867 0.329 11.066 H 1 ZIN1 0.000

48 H16 -14.081 1.175 12.517 H 1 ZIN1 0.000

49 H17 -13.196 -0.191 10.370 H 1 ZIN1 0.000

50 H18 -12.288 0.611 11.795 H 1 ZIN1 0.000

51 H19 -11.424 1.669 10.464 H 1 ZIN1 0.000

52 H20 -13.496 3.442 9.570 H 1 ZIN1 0.000

53 H21 -10.094 3.394 7.899 H 1 ZIN1 0.000

54 H22 -10.276 2.394 9.359 H 1 ZIN1 0.000

55 H23 -9.384 4.476 9.058 H 1 ZIN1 0.000

56 H24 -9.695 3.425 11.026 H 1 ZIN1 0.000

57 H25 -9.115 6.638 10.688 H 1 ZIN1 0.000

58 H26 -10.809 7.180 10.622 H 1 ZIN1 0.000

59 H27 -10.045 7.989 8.172 H 1 ZIN1 0.000

60 H28 -8.455 8.041 8.968 H 1 ZIN1 0.000

61 H29 -9.377 9.477 10.751 H 1 ZIN1 0.000

62 H30 -10.988 9.396 10.000 H 1 ZIN1 0.000

63 H31 -8.503 10.693 9.158 H 1 ZIN1 0.000

64 H32 -10.174 9.941 7.160 H 1 ZIN1 0.000

65 H33 -8.389 9.616 7.575 H 1 ZIN1 0.000

66 H34 -9.719 11.380 6.033 H 1 ZIN1 0.000

67 H35 -7.902 11.156 6.372 H 1 ZIN1 0.000

68 H36 -9.697 13.135 6.711 H 1 ZIN1 0.000

69 H37 -7.878 13.034 7.096 H 1 ZIN1 0.000

70 H38 -10.127 13.456 8.515 H 1 ZIN1 0.000

71 H39 -8.338 13.369 9.027 H 1 ZIN1 0.000

72 H40 -8.729 11.806 10.131 H 1 ZIN1 0.000

73 H41 -10.544 12.027 9.780 H 1 ZIN1 0.000

74 H42 -13.151 3.665 8.438 H 1 ZIN1 0.000

75 H44 -11.626 5.315 7.035 H 1 ZIN1 0.000

76 H45 -14.220 4.426 6.357 H 1 ZIN1 0.000

77 H46 -14.414 2.905 7.239 H 1 ZIN1 0.000

78 H47 -15.996 5.695 6.320 H 1 ZIN1 0.000

79 H48 -14.383 6.591 6.565 H 1 ZIN1 0.000

80 H49 -15.291 8.297 6.330 H 1 ZIN1 0.000

81 H50 -16.855 7.389 5.882 H 1 ZIN1 0.000

82 H43 -10.521 5.865 8.261 H 1 ZIN1 0.162

@<TRIPOS>BOND

1 1 33 1

2 1 34 1

3 1 35 1

4 1 2 1

5 2 32 1

6 2 36 1

7 2 3 1

8 3 37 1

9 3 38 1

10 3 4 1

11 4 39 1

12 4 40 1

13 4 5 1

14 5 31 1

15 5 41 1

16 5 6 1

17 6 7 1

18 6 30 1

19 6 42 1

20 7 8 1

21 7 43 1

22 8 9 1

23 8 13 1

24 8 44 1

25 9 10 1

26 9 45 1

27 9 46 1

28 10 11 1

29 10 47 1

30 10 48 1

31 11 12 1

32 11 49 1

33 11 50 1

34 12 13 1

35 12 51 1

36 13 14 1

37 13 52 1

38 14 15 1

39 14 28 1

40 15 16 1

41 15 53 1

42 15 54 1

43 16 17 1

44 16 18 1

45 16 55 1

46 17 56 1

47 18 19 1

48 18 82 1

49 19 20 1

50 19 57 1

51 19 58 1

52 20 21 1

53 20 59 1

54 20 60 1

55 21 22 1

56 21 61 1

57 21 62 1

58 22 23 1

59 22 27 1

60 22 63 1

61 23 64 1

62 23 65 1

63 23 24 1

64 24 66 1

65 24 67 1

66 24 25 1

67 25 68 1

68 25 69 1

69 25 26 1

70 26 70 1

71 26 71 1

72 26 27 1

73 27 72 1

74 27 73 1

75 28 29 1

76 28 30 1

77 28 74 1

78 29 75 1

79 30 76 1

80 30 77 1

81 31 78 1

82 31 79 1

83 31 32 1

84 32 80 1

85 32 81 1

@<TRIPOS>SUBSTRUCTURE

1 ZIN1 1 GROUP 1 d ZIN

**ZINC000101867325.mol2**

@<TRIPOS>MOLECULE

ZIN1

72 76 1

SMALL

USER_CHARGES

@<TRIPOS>ATOM

1 C1 -16.594 -6.169 12.390 C.3 1 ZIN1 0.021

2 C2 -16.819 -4.775 12.978 C.3 1 ZIN1 0.095

3 N3 -15.636 -4.375 13.753 N.3 1 ZIN1 -0.240

4 C4 -15.928 -4.301 15.099 C.3 1 ZIN1 0.122

5 N5 -16.999 -4.899 15.598 N.3 1 ZIN1 -0.248

6 C6 -17.286 -4.840 16.885 C.3 1 ZIN1 0.118

7 C7 -16.484 -4.152 17.776 C.3 1 ZIN1 0.020

8 C8 -15.351 -3.503 17.311 C.3 1 ZIN1 0.022

9 C9 -15.071 -3.578 15.949 C.3 1 ZIN1 0.072

10 C10 -13.894 -2.911 15.360 C.3 1 ZIN1 0.256

11 O11 -12.961 -2.545 16.050 O.3 1 ZIN1 -0.267

12 N12 -13.896 -2.723 14.018 N.3 1 ZIN1 -0.334

13 C13 -15.068 -3.126 13.236 C.3 1 ZIN1 0.097

14 C14 -14.651 -3.324 11.775 C.3 1 ZIN1 0.068

15 C15 -14.030 -2.028 11.247 C.3 1 ZIN1 0.130

16 N16 -14.980 -0.928 11.455 N.3 1 ZIN1 -0.348

17 C17 -15.350 -0.135 10.429 C.3 1 ZIN1 0.256

18 O18 -14.983 -0.394 9.299 O.3 1 ZIN1 -0.267

19 C19 -16.199 1.044 10.678 C.3 1 ZIN1 0.063

20 C20 -15.762 2.335 10.289 C.3 1 ZIN1 0.101

21 N21 -14.581 2.512 9.692 N.3 1 ZIN1 -0.256

22 C22 -14.203 3.722 9.343 C.3 1 ZIN1 0.136

23 C23 -15.025 4.824 9.590 C.3 1 ZIN1 0.136

24 N24 -16.190 4.673 10.176 N.3 1 ZIN1 -0.257

25 C25 -16.597 3.453 10.540 C.3 1 ZIN1 0.086

26 C26 -17.840 3.260 11.169 C.3 1 ZIN1 0.034

27 C27 -18.238 2.009 11.533 C.3 1 ZIN1 0.003

28 C28 -17.436 0.901 11.290 C.3 1 ZIN1 0.020

29 C29 -15.522 -0.708 12.803 C.3 1 ZIN1 0.130

30 C30 -16.126 -2.021 13.312 C.3 1 ZIN1 0.068

31 H01 -15.603 -6.525 12.670 H 1 ZIN1 0.000

32 H02 -17.348 -6.854 12.778 H 1 ZIN1 0.000

33 H03 -16.669 -6.122 11.304 H 1 ZIN1 0.000

34 H04 -17.692 -4.792 13.630 H 1 ZIN1 0.000

35 H05 -16.984 -4.061 12.171 H 1 ZIN1 0.000

36 H06 -16.766 -4.902 14.746 H 1 ZIN1 0.000

37 H07 -17.795 -4.513 15.110 H 1 ZIN1 0.000

38 H08 -17.150 -5.874 17.201 H 1 ZIN1 0.000

39 H09 -18.279 -4.396 16.959 H 1 ZIN1 0.000

40 H10 -16.085 -4.936 18.420 H 1 ZIN1 0.000

41 H11 -17.111 -3.397 18.251 H 1 ZIN1 0.000

42 H12 -14.522 -4.024 17.790 H 1 ZIN1 0.000

43 H13 -15.453 -2.448 17.565 H 1 ZIN1 0.000

44 H14 -14.606 -3.282 16.889 H 1 ZIN1 0.000

45 H15 -13.058 -2.588 15.981 H 1 ZIN1 0.000

46 H16 -12.661 -3.275 16.597 H 1 ZIN1 0.000

47 H17 -15.526 -3.580 11.177 H 1 ZIN1 0.000

48 H18 -13.922 -4.131 11.710 H 1 ZIN1 0.000

49 H19 -13.809 -2.130 10.184 H 1 ZIN1 0.000

50 H20 -13.104 -1.820 11.783 H 1 ZIN1 0.000

51 H21 -15.536 0.859 10.022 H 1 ZIN1 0.000

52 H22 -15.029 0.398 8.758 H 1 ZIN1 0.000

53 H23 -15.480 0.257 10.905 H 1 ZIN1 0.000

54 H24 -14.944 1.671 10.010 H 1 ZIN1 0.000

55 H25 -13.877 2.155 10.322 H 1 ZIN1 0.000

56 H26 -14.190 3.668 8.254 H 1 ZIN1 0.000

57 H27 -13.260 3.915 9.855 H 1 ZIN1 0.000

58 H28 -15.298 5.152 8.587 H 1 ZIN1 0.000

59 H29 -14.449 5.509 10.212 H 1 ZIN1 0.000

60 H30 -16.172 5.238 11.013 H 1 ZIN1 0.000

61 H31 -17.239 4.322 10.682 H 1 ZIN1 0.000

62 H32 -18.555 3.531 10.392 H 1 ZIN1 0.000

63 H33 -17.830 3.863 12.077 H 1 ZIN1 0.000

64 H34 -19.093 1.832 10.880 H 1 ZIN1 0.000

65 H35 -18.408 2.039 12.609 H 1 ZIN1 0.000

66 H36 -17.174 0.566 12.293 H 1 ZIN1 0.000

67 H37 -18.018 0.226 10.663 H 1 ZIN1 0.000

68 H38 -16.290 0.065 12.772 H 1 ZIN1 0.000

69 H40 -14.723 -0.387 13.472 H 1 ZIN1 0.000

70 H41 -16.452 -1.897 14.345 H 1 ZIN1 0.000

71 H42 -16.982 -2.292 12.695 H 1 ZIN1 0.000

72 H39 -13.128 -2.325 13.579 H 1 ZIN1 0.164

@<TRIPOS>BOND

1 1 31 1

2 1 32 1

3 1 33 1

4 1 2 1

5 2 34 1

6 2 35 1

7 2 3 1

8 3 4 1

9 3 13 1

10 4 5 1

11 4 9 1

12 4 36 1

13 5 6 1

14 5 37 1

15 6 7 1

16 6 38 1

17 6 39 1

18 7 8 1

19 7 40 1

20 7 41 1

21 8 9 1

22 8 42 1

23 8 43 1

24 9 10 1

25 9 44 1

26 10 11 1

27 10 12 1

28 10 45 1

29 11 46 1

30 12 13 1

31 12 72 1

32 13 14 1

33 13 30 1

34 14 15 1

35 14 47 1

36 14 48 1

37 15 16 1

38 15 49 1

39 15 50 1

40 16 17 1

41 16 29 1

42 17 18 1

43 17 19 1

44 17 51 1

45 18 52 1

46 19 20 1

47 19 28 1

48 19 53 1

49 20 21 1

50 20 54 1

51 21 22 1

52 21 55 1

53 22 23 1

54 22 56 1

55 22 57 1

56 23 58 1

57 23 59 1

58 23 24 1

59 24 60 1

60 20 25 1

61 24 25 1

62 25 61 1

63 25 26 1

64 26 62 1

65 26 63 1

66 26 27 1

67 27 64 1

68 27 65 1

69 27 28 1

70 28 66 1

71 28 67 1

72 29 30 1

73 29 68 1

74 29 69 1

75 30 70 1

76 30 71 1

@<TRIPOS>SUBSTRUCTURE

1 ZIN1 1 GROUP 1 d ZIN

**ZINC000101881326.mol2**

@<TRIPOS>MOLECULE

ZIN1

74 78 1

SMALL

USER_CHARGES

@<TRIPOS>ATOM

1 C1 -9.187 12.985 7.263 C.3 1 ZIN1 0.210

2 O2 -8.677 12.141 8.298 O.3 1 ZIN1 -0.353

3 C3 -8.975 10.818 8.218 C.3 1 ZIN1 0.101

4 C4 -8.599 10.091 7.096 C.3 1 ZIN1 0.040

5 C5 -8.899 8.749 7.009 C.3 1 ZIN1 0.016

6 C6 -9.582 8.116 8.053 C.3 1 ZIN1 -0.008

7 C7 -9.902 6.685 7.962 C.3 1 ZIN1 0.066

8 N8 -9.207 5.938 7.144 N.3 1 ZIN1 -0.063

9 C9 -9.559 4.635 6.992 C.3 1 ZIN1 0.767

10 N10 -8.930 3.625 6.447 N.3 1 ZIN1 0.067

11 N11 -9.641 2.554 6.539 N.3 1 ZIN1 -0.008

12 N12 -10.763 2.799 7.136 N.3 1 ZIN1 -0.029

13 N13 -10.759 4.154 7.447 N.3 1 ZIN1 -0.066

14 C14 -11.753 5.001 8.100 C.3 1 ZIN1 0.174

15 C16 -12.531 4.190 9.105 C.3 1 ZIN1 0.034

16 C17 -13.871 3.997 9.106 C.3 1 ZIN1 0.138

17 N19 -14.173 3.213 10.173 N.3 1 ZIN1 -0.222

18 C20 -15.452 2.773 10.540 C.3 1 ZIN1 0.060

19 C21 -15.720 1.413 10.628 C.3 1 ZIN1 0.031

20 C22 -16.981 0.981 10.990 C.3 1 ZIN1 0.002

21 C23 -17.976 1.902 11.264 C.3 1 ZIN1 0.000

22 C24 -17.712 3.256 11.178 C.3 1 ZIN1 0.002

23 C25 -16.451 3.695 10.821 C.3 1 ZIN1 0.031

24 N26 -12.977 2.922 10.841 N.3 1 ZIN1 -0.171

25 C27 -11.995 3.502 10.198 C.3 1 ZIN1 0.063

26 C28 -10.538 3.446 10.577 C.3 1 ZIN1 0.073

27 C29 -11.010 6.138 8.818 C.3 1 ZIN1 0.094

28 C30 -9.960 8.850 9.184 C.3 1 ZIN1 0.053

29 C31 -9.652 10.193 9.265 C.3 1 ZIN1 0.102

30 O32 -10.017 10.909 10.361 O.3 1 ZIN1 -0.353

31 C33 -10.817 10.239 11.337 C.3 1 ZIN1 0.210

32 Cl18 -15.001 4.635 7.951 Cl 1 ZIN1 -0.062

33 H01 -10.085 13.491 7.617 H 1 ZIN1 0.000

34 H02 -8.435 13.727 6.994 H 1 ZIN1 0.000

35 H03 -9.432 12.382 6.389 H 1 ZIN1 0.000

36 H04 -9.907 10.367 7.878 H 1 ZIN1 0.000

37 H05 -7.510 10.100 7.147 H 1 ZIN1 0.000

38 H06 -9.065 10.574 6.237 H 1 ZIN1 0.000

39 H07 -7.919 8.272 7.025 H 1 ZIN1 0.000

40 H08 -9.504 8.619 6.112 H 1 ZIN1 0.000

41 H09 -9.766 7.062 8.259 H 1 ZIN1 0.000

42 H10 -10.224 5.655 7.806 H 1 ZIN1 0.000

43 H11 -9.257 6.369 6.232 H 1 ZIN1 0.000

44 H12 -8.868 5.466 6.852 H 1 ZIN1 0.000

45 H13 -8.755 3.827 5.473 H 1 ZIN1 0.000

46 H14 -9.829 2.199 5.612 H 1 ZIN1 0.000

47 H15 -11.538 2.583 6.525 H 1 ZIN1 0.000

48 H16 -12.448 5.406 7.364 H 1 ZIN1 0.000

49 H17 -11.971 4.789 8.387 H 1 ZIN1 0.000

50 H18 -14.576 4.404 8.382 H 1 ZIN1 0.000

51 H19 -15.455 3.050 9.486 H 1 ZIN1 0.000

52 H20 -15.095 1.087 11.459 H 1 ZIN1 0.000

53 H21 -15.509 0.986 9.648 H 1 ZIN1 0.000

54 H22 -16.813 0.500 11.954 H 1 ZIN1 0.000

55 H23 -17.346 0.356 10.175 H 1 ZIN1 0.000

56 H24 -18.166 1.753 12.327 H 1 ZIN1 0.000

57 H25 -18.794 1.697 10.573 H 1 ZIN1 0.000

58 H26 -17.811 3.595 12.209 H 1 ZIN1 0.000

59 H27 -18.407 3.666 10.446 H 1 ZIN1 0.000

60 H28 -16.617 4.186 9.862 H 1 ZIN1 0.000

61 H29 -16.088 4.314 11.641 H 1 ZIN1 0.000

62 H30 -13.020 3.272 11.788 H 1 ZIN1 0.000

63 H31 -10.936 3.427 10.446 H 1 ZIN1 0.000

64 H32 -9.990 4.210 10.025 H 1 ZIN1 0.000

65 H33 -10.433 3.626 11.647 H 1 ZIN1 0.000

66 H34 -10.136 2.463 10.333 H 1 ZIN1 0.000

67 H35 -11.719 6.941 9.021 H 1 ZIN1 0.000

68 H36 -10.588 5.757 9.748 H 1 ZIN1 0.000

69 H37 -9.366 8.406 9.983 H 1 ZIN1 0.000

70 H38 -11.044 8.769 9.266 H 1 ZIN1 0.000

71 H39 -8.702 9.901 9.712 H 1 ZIN1 0.000

72 H40 -11.792 10.005 10.909 H 1 ZIN1 0.000

73 H41 -10.323 9.316 11.641 H 1 ZIN1 0.000

74 H42 -10.948 10.884 12.206 H 1 ZIN1 0.000

@<TRIPOS>BOND

1 1 33 1

2 1 34 1

3 1 35 1

4 1 2 1

5 2 3 1

6 3 29 1

7 3 36 1

8 3 4 1

9 4 37 1

10 4 38 1

11 4 5 1

12 5 39 1

13 5 40 1

14 5 6 1

15 6 28 1

16 6 41 1

17 6 7 1

18 7 8 1

19 7 27 1

20 7 42 1

21 8 9 1

22 8 43 1

23 9 10 1

24 9 13 1

25 9 44 1

26 10 11 1

27 10 45 1

28 11 12 1

29 11 46 1

30 12 13 1

31 12 47 1

32 13 14 1

33 14 15 1

34 14 27 1

35 14 48 1

36 15 16 1

37 15 25 1

38 15 49 1

39 16 32 1

40 16 50 1

41 16 17 1

42 17 18 1

43 18 19 1

44 18 23 1

45 18 51 1

46 19 52 1

47 19 53 1

48 19 20 1

49 20 54 1

50 20 55 1

51 20 21 1

52 21 56 1

53 21 57 1

54 21 22 1

55 22 58 1

56 22 59 1

57 22 23 1

58 23 60 1

59 23 61 1

60 17 24 1

61 24 62 1

62 24 25 1

63 25 26 1

64 25 63 1

65 26 64 1

66 26 65 1

67 26 66 1

68 27 67 1

69 27 68 1

70 28 69 1

71 28 70 1

72 28 29 1

73 29 30 1

74 29 71 1

75 30 31 1

76 31 72 1

77 31 73 1

78 31 74 1

@<TRIPOS>SUBSTRUCTURE

1 ZIN1 1 GROUP 1 d ZIN
